# Structural and optical control through anion and cation exchange processes for Sn-halide perovskite nanostructures†

**DOI:** 10.1039/d3nr06075f

**Published:** 2024-02-06

**Authors:** Kushagra Gahlot, Julius Meijer, Loredana Protesescu

**Affiliations:** a Zernike Institute for Advanced Materials, University of Groningen Nijenborgh 4 Groningen 9747AG The Netherlands l.protesescu@rug.nl

## Abstract

Metal halide perovskite nanostructures, characterized by their ionic nature, present a compelling avenue for the tunability of dimensions and band gaps through facile compositional transformations involving both cationic and anionic exchange reactions. While post-synthetic ion-exchange processes have been extensively explored in Pb-halide perovskite nanocrystals, the inherent instability of Sn^2+^ has limited the exploration of such processes in Sn-halide perovskite nanostructures. In this study, we present a straightforward cation exchange process wherein 2D [R–NH_3_]_2_SnX_4_ Ruddlesden–Popper (RP) nanostructures with *n* = 1 transition to 3D ASnX_3_ nanocrystals at room temperature with the addition of A-cation oleate. In addition, anion exchange processes have been demonstrated for both 2D [R–NH_3_]_2_SnX_4_ RP nanostructures and 3D nanocrystals, showcasing transitions between iodide and bromide counterparts. Furthermore, we have fabricated a thin film of 2D [R–NH_3_]_2_SnX_4_ RP nanostructures for cation exchange, wherein A-cation diffusion through a liquid–solid interface facilitates the transformation into a 3D ASnX_3_ crystal. This investigation underscores the versatility of ion exchange processes in engineering the composition of Sn-halide perovskite nanostructures and, consequently, modulating their optical properties.

## Abstract 2

Metal halide perovskite nanostructures, characterized by their ionic nature, present a compelling avenue for the tunability of dimensions and band gaps through compositional transformations involving both cationic and anionic exchange reactions.

## Graphical abstract

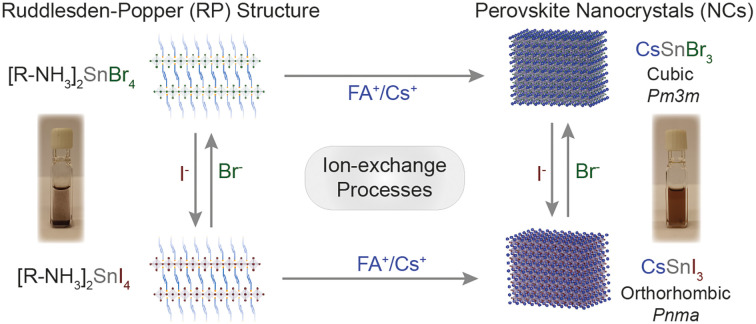

## Introduction

Ion exchange processes in semiconductor nanocrystals (NCs) have emerged as potent chemical methods to easily modulate their composition, crystal structure, and hence, properties, forming new high-quality colloidal nanomaterials inaccessible *via* the conventional direct synthetic methodology.^4–7^ The conventional semiconductor NCs (quantum dots, QDs, III–V, II–VI, and IV–VI) as well as metal halide perovskite NCs demonstrate these ion exchange capabilities for both cations and anions while maintaining the structural and morphological integrity of the NCs.^8^ The ion exchange processes in QDs need various reaction conditions, depending on the ions to be exchanged and the nature of their bonding. For example, to form III–V, Alivisatos and co-workers reported cation exchange of Cd^2+^ in Cd_2_As_3_ NCs 4.55 nm *via* In^3+^/Ga^3+^ at a temperature of 270 °C.^9^ Moreover, for II–VI NCs, an excess concentration of the exchange cation can drive the reaction at room temperature completely exchanging Ag^+^ in 4.2 nm CdSe NCs to form Ag_2_Se NCs and *vice versa*.^5,10,11^ The diffusion of the cation into the crystal structure while keeping the anion sub-lattice intact has led to high quality NCs in various QDs while the anion exchange processes have usually yielded poor-quality materials owing to the low mobility of anions.^4,12^

In halide perovskite NCs with the chemical formula AMX_3_, where M = Pb^2+^ and Sn^2+^ and halide X = Cl^−^, Br^−^, and I^−^, with the A cation being Cs^+^ or FA^+^, the ionic nature of bonding facilitates these exchange processes under relatively milder reaction conditions.^13^ Nedelcu *et al.*^6^ and Akkerman *et al.*^14^ studied the post-synthetic anion exchange in Pb-halide perovskite NCs at room temperature to achieve compositional tunability (I, Br, and Cl) and hence, emission tunability over the full visible region.^6^ Following these reports, for Pb-halide perovskite NCs, cation (A-site) exchange was also successfully achieved at room temperature in both bulk thin films and NCs (in solution and thin films).^14–20^ For example, in mixed Pb–Sn perovskite bulk thin films, the exchange of longer organic cations (such as phenyl ethylammonium) with small organic cations (such as formamidinium) by just dipping the thin film in the exchange cation solution was reported.^16^ In this case, the A cation exchange also led to a change in the dimensionality of the perovskite structure from 2D to 3D.

While reports are abundant for Pb-based perovskites in all forms, the Sn halide perovskite chemistry has remained unexplored. This is majorly due to the lack of synthetic reports on Sn-halide perovskite NCs owing to their ambient environment instability, a result of the oxidation process (Sn^2+^ → Sn^4+^ + 2e^−^) impairing the existence of the desired ASnX_3_ chemical composition.

**Scheme 1:**
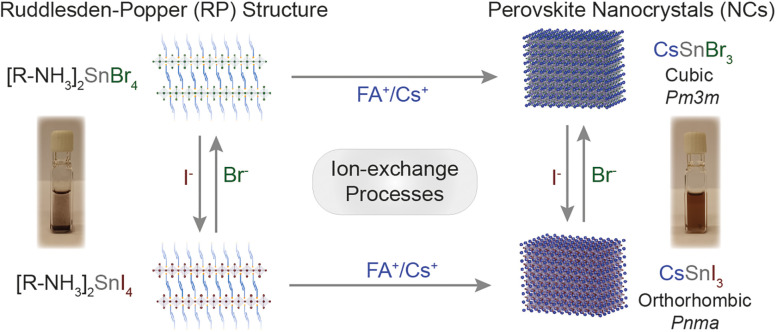
Overview of the ion exchange processes successfully achieved to Sn-halide perovskite nanostructures.

In this work, we took advantage of the earlier reported synthesis of high-quality CsSnI_3_ NCs,^21^ and demonstrated the cation exchange and anion processes for the Sn-halide perovskite nanostructures of the 2D [R–NH_3_]_2_SnX_4_ Ruddlesden–Popper (RP) perovskite as well as 3D ASnX_3_ NCs. Scheme 1 outlines the successfully demonstrated ion exchange processes in these materials. We showed that the cation exchange of [R–NH_3_]^+^ in 2D [R–NH_3_]_2_SnX_4_ with the Cs^+^/FA^+^ cation increases the dimensionality of the nanostructures from 2D to 3D at room temperature. The cation exchange reactions can be performed both in solution and in thin films taking advantage of the solid–liquid interface. Also, directly synthesized 2D [R–NH_3_]_2_SnX_4_ RP nanostructures and 3D CsSnX_3_ NCs can exchange the anions at room temperature, specifically bromide to iodide and *vice versa* by utilizing benzoyl halides (BzX) as the anion source. The anion exchange on directly synthesized CsSnI_3_ and CsSnBr_3_ NCs was performed to achieve partial and complete exchange for bromide and iodide, respectively. The utilization of excess benzoyl iodide (BzI) led to the oxidation of Sn^2+^, resulting in the double perovskite structure Cs_2_SnI_6_.

## Results and discussion

### 2D oleyl ammonium-based RP *n* = 1 Sn-halide perovskite nanostructures

**Fig. 1:**
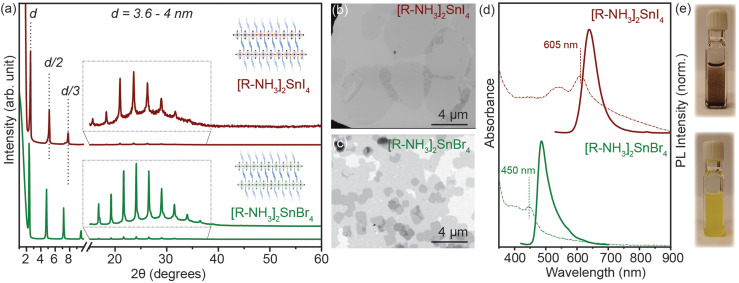
(a) Powder XRD pattern of the 2D [R–NH_3_]_2_SnI_4_ (brown) and [R–NH_3_]_2_SnBr_4_ (green) RP nanostructures respectively. (b and c) SEM Images of the 2D [R–NH_3_]_2_SnI_4_ (brown) and [R–NH_3_]_2_SnBr_4_ (green) RP nanostructures showing the nanosheet morphology. (d) UV–visible absorption (dotted line) and PL (solid line) spectra of the 2D [R–NH_3_]_2_SnI_4_ (brown) and [R–NH_3_]_2_SnBr_4_ (green) RP nanostructures, respectively. (e) Photographs of 2D [R–NH_3_]_2_SnI_4_ (dark brown suspension) and [R–NH_3_]_2_SnBr_4_ (yellow suspension) RP nanostructures in visible light.

2D RP Sn-halide perovskite structures have the general chemical formula R_2_A_*n*−1_Sn_*n*_X_3*n*+1_ (*n* = 1); here R is a monovalent long-chain organic cation and A is a small organic or inorganic cation, in our case cesium (Cs^+^) and formamidinium (FA^+^). For this study, we employed oleyl ammonium (C_18_H_38_N^+^–[R–NH_3_]^+^) to synthesize *n* = 1 2D [R–NH_3_]_2_SnX_4_ RP nanostructures. SnX_2_ salts were complexed with oleyl amine (OLA) and oleic acid (OA) in a non-coordinating solvent, *i.e.* octadecene (ODE), to form a transparent coloured solution above 160 °C. This transparent solution self-assembled to form *n* = 1 2D [R–NH_3_]_2_SnX_4_ RP structures when cooled to room temperature with the help of ice-water (detailed description in the Experimental section). Fig. 1(a) shows the powder X-ray diffraction (XRD) pattern of the 2D [R–NH_3_]_2_SnBr_4_ (green) and [R–NH_3_]_2_SnI_4_ (brown) nanosheets with an aspect ratio of 6 : 5 in both cases. As [R–NH_3_]^+^ has a C_18_ chain, the *n* = 1 RP nanosheets both with I and Br had *d*-spacings as high as 3.6 to 4 nm, which were calculated by using the 2*θ* periodicity of diffraction peaks observed at low angles of the diffraction pattern.^22^ These 2D RP nanosheets are highly ordered, identified by the presence of secondary interference peaks at higher 2*θ* degrees (inset in Fig. 1(a)).^23^ We also observed that the *d*-spacings are dependent on the quenching rate of the reaction mixture as the organic moiety is flexible in nature as shown in Fig. S1.† We further analyzed the morphology and size of these 2D RP nanostructures using scanning electron microscopy (SEM) performed in the transmission mode at 18 kV as shown in Fig. 1(b) and (c), which showed the presence of nanosheets (≈4–6 μm in length), with an in-plane aspect ratio of 15 to 20 for iodide and bromide counterparts, respectively. Since these 2D nanostructures are a monolayer of [SnX_6_]^4−^ octahedra inter-coordinated with [R–NH_3_]^+^, they showed a strong quantum confinement effect which can be observed from their optical properties. Fig. 1(d) shows the UV–visible absorption (dotted line) and photoluminescence (PL) (solid line) spectra for 2D [R–NH_3_]_2_SnBr_4_ (green) and [R–NH_3_]_2_SnI_4_ (brown) RP nanosheets. The absorption spectra showed a narrow excitonic peak at 450 nm for 2D [R–NH_3_]_2_SnBr_4_ and at 605 nm for 2D [R–NH_3_]_2_SnI_4_ with a relatively sharp PL peak at 488 and 638 nm, respectively (FWHM of 0.2 eV and 0.15 eV, respectively). These self-assembled 2D RP Sn-halide perovskite nanostructures are not colloidally stable as can be observed *via* the snapshots in Fig. 1(e), and from the scattering observed in the UV–visible spectroscopy absorption spectra.

### Cation exchange in a 2D RP perovskite suspension

**Fig. 2:**
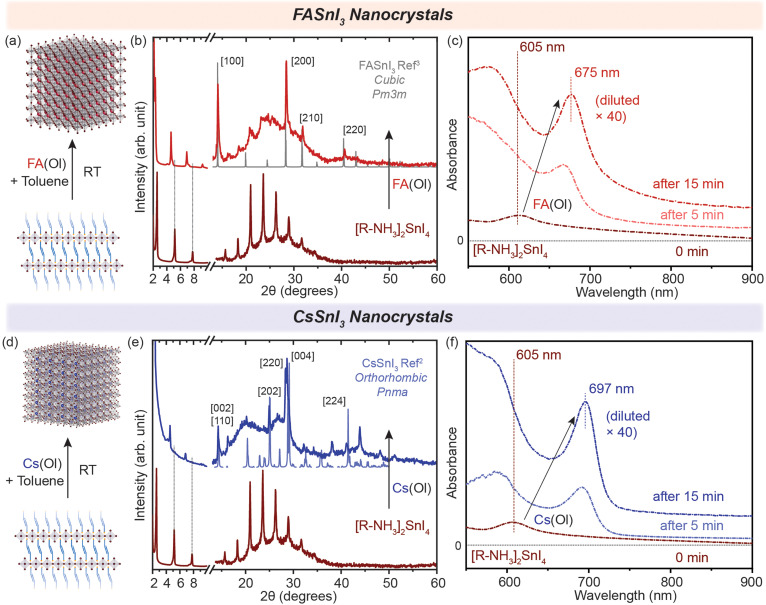
(a and d) Schematic representation of the conversion of 2D [R–NH_3_]_2_SnI_4_ into 3D FASnI_3_ and CsSnI_3_ NCs, respectively. (b and e) Powder XRD pattern of the converted 3D FASnI_3_ (red) and CsSnI_3_ (blue) NCs from 2D [R–NH_3_]_2_SnI_4_ nanosheets, respectively. (c and f) *In situ* UV–visible absorption spectra showing the formation of 3D FASnI_3_ (red) and CsSnI_3_ (blue) NCs from the 2D [R–NH_3_]_2_SnI_4_ RP nanosheet structure with the addition of A cation. The XRD references are adapted from ref. 3 for FASnI_3_ (cubic, *Pm*3*m*) and ref. 2 for CsSnI_3_ (orthorhombic, *Pnma*).

Generally, in metal halide perovskites, the cation exchange process has a high activation energy of ≈0.65 eV for exchange between 3D cations (Cs^+^ and FA^+^). For larger cations, this can even cause the disintegration of lattice structures undergoing exchange reactions.^24^ Nonetheless, post-synthetic cation exchange processes have been demonstrated in Pb-halide perovskite NCs and in thin films under precisely controlled physical and chemical parameters,^24–27^ but not yet fully understood for Sn halide perovskite nanostructures. We further probed how efficient this process can be for our synthesized nanostructures. Oleate complexes of an organic cation, *i.e.* FA^+^, and an inorganic cation, *i.e.* Cs^+^, were utilized for the cation exchange reaction to obtain 2D [R–NH_3_]_2_SnBr_4_ (green in Fig. 1) and [R–NH_3_]_2_SnI_4_ (brown in Fig. 1) RP perovskite nanostructures. In short, a colloidal suspension of 2D [R–NH_3_]_2_SnX_4_ RP perovskite nanostructures was injected with the FA(Ol) and Cs(Ol) precursors under vigorous stirring at room temperature; the solution became homogeneous and colloidally stable. These cation-exchanged solutions were then purified following the protocol mentioned in the Experimental section. We observed that cation exchange with a small-sized cation, which can form the 3D perovskite structure, increased the dimensionality of the perovskite from 2D to 3D in the form of NCs because of the remaining oleate in the system. The oleate together with extra R–NH_3_^+^ will ensure the colloidal stability of the newly formed NCs. We tracked this transformation using absorption and PL spectroscopy (Fig. 2(c) and (f)). Here, we noted a red shift of the excitonic peak from 605 nm to 675 nm and from 450 nm to 488 nm for both the halide systems in FASnX_3_ NCs.

The cation exchange process is illustrated in Fig. 2(a) and (d). In the case of the X = I system, the XRD pattern in Fig. 2(b) and (e) clearly shows the presence of diffraction peaks at higher 2*θ* values, which correspond to the FASnI_3_ and CsSnI_3_ NCs in the cubic crystal structure (*Pm*3*m*) and the orthorhombic crystal structure (*Pnma*), respectively. The diffraction peaks at lower angles and secondary interferences belonging to the 2D [R–NH_3_]_2_SnI_4_ (brown) RP perovskite diminished, showing the conversion of 2D [R–NH_3_]_2_SnI_4_ into both FASnI_3_ and CsSnI_3_. Similarly, for the cation exchange in 2D [R–NH_3_]_2_SnBr_4_ (green), the injection of the FA(Ol) and Cs(Ol) precursor solution led to the formation of FASnBr_3_ and CsSnBr_3_ NCs, which crystallized in the cubic perovskite crystal structure (*Pm*3*m*) as can be observed in Fig. S2(b) and (e)† with the presence of 2D [R–NH_3_]_2_SnBr_4_ in the Cs case. Note that the diffraction measurements were performed under an inert atmosphere using a dome that cannot be revolved; therefore the relative intensities of the diffraction peaks toward a certain lattice plane are preferred over others. Fig. S3† shows the SEM images demonstrating the formation of 3D FASnI_3_ NCs with an average edge length of 15–20 nm as an example. This change in the dimensionality of the Sn-halide perovskite nanostructures from 2D to 3D is better represented by their optical properties. Fig. 2(c) shows the evolution of UV–visible absorption spectra with the addition of FA(Ol) in a non-colloidal suspension of 2D [R–NH_3_]_2_SnI_4_. We observed that the excitonic peak began to red shift from 605 nm and remained at 675 nm after 15 min. At that point, the solution became colloidally stable and saturated the detector. Hence, we used a 40 times diluted solution to perform the absorption measurements. These optical features are in a similar range as reported earlier for FASnI_3_ NCs.^28,29^ For Cs(Ol) addition, the excitonic peak remained at 697 nm after 15 min for half the concentration when compared to FA(Ol) (Fig. 2(f)). Fig. S2(c) and (e)† plot the temporal change of UV–visible spectra for the bromide case, where a similar red shift was observed; in the case of FA(Ol), the peak remained at 488 nm and in the case of Cs(Ol), the peak remained at 530 nm after 15 min for the same concentration of the cation. The bulk band gap for FASnBr_3_ and CsSnBr_3_ for the cubic perovskite crystal structure (*Pm*3*m*) was previously reported to be 2.4 eV (≈516 nm) and 1.92 (≈645 nm).^30–32^ Thus, our observations are in good agreement with the formed nanostructures being within the confinement range of these materials. We also kept the cation exchange reaction for extended times up to 20 h (Fig. S4†), which led to more bulk-like structures. Furthermore, it was essential to know the effect of the ratios of FA(Ol) and Cs(Ol) with respect to 2D [R–NH_3_]_2_SnX_4_ RP perovskite nanostructures on the cation exchange process and the final product obtained.

**Fig. 3:**
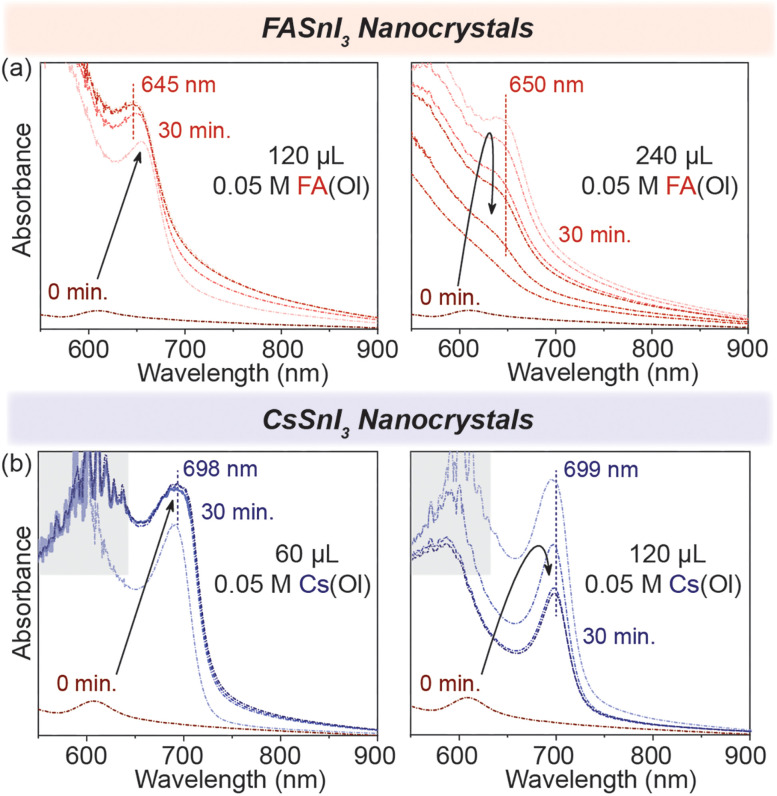
Concentration dependence *in situ* UV–visible absorption spectroscopy. (a) The formation of the 3D FASnI_3_ (red) NCs with the addition of FA(Ol) in the 2D [R–NH_3_]_2_SnI_4_ RP structures for 30 min. (b) The formation of the 3D CsSnI_3_ (blue) NCs with the addition of Cs(Ol) in the 2D [R–NH_3_]_2_SnI_4_ RP structures for 30 min. The grey area highlights the detector saturation region in the above plots.

To test this, we started by adding 120 μL of 0.05 M FA(Ol), and within 30 min, we observed that the excitonic peak remained stable at 645 nm (Fig. 3(a)). However, when the ratio of FA : [R–NH_3_]_2_SnI_4_ was increased, and we doubled the volume of FA(Ol) added to 240 μL of 0.05 M solution, the wavelength of the excitonic peak quickly increased from 605 nm to be stabilized as a broad feature at 650 nm. We concluded that the concentration and volume of the FA(Ol) solution dictate the steady transformation into 3D NCs over a period of time. For the chemical transformation of [R–NH_3_]_2_SnI_4_ nanosheets into CsSnI_3_ NCs, similar evolution was observed in the absorption spectra, with the excitonic peak stable at 699 nm, as displayed in Fig. 3(b). Furthermore, the bromide counterparts demonstrated similar evolution of absorbance spectra with excitonic peaks stabilizing at 540 nm and 600 nm for FASnBr_3_ and CsSnBr_3_, respectively (Fig. S5†). For the cation exchange from [R–NH_3_]^+^ to FA^+^ or Cs^+^, three simultaneous processes are involved in this process, namely, the cation exchange chemical reaction, a structural reconstruction that is not thermodynamically favorable, and a morphology change. Therefore, the cation exchange reactions are highly sensitive to the reaction time and concentration and require a precise supply of exchange cations to recrystallize into 3D halide perovskite NCs.^33,34^

### Cation exchange in 2D RP perovskite thin films

**Fig. 4:**
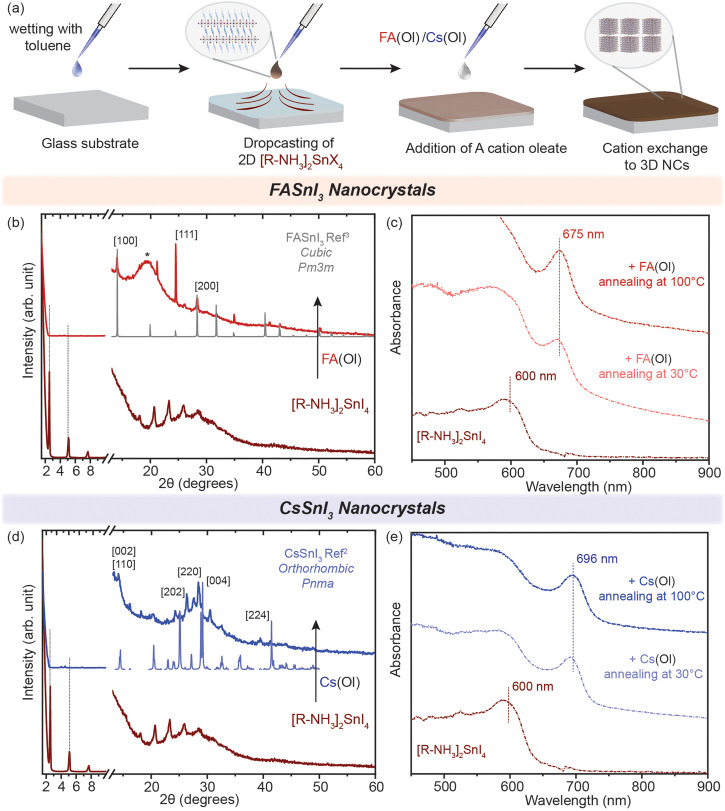
Cation exchange in thin films. (a) Schematic of the methodology utilized to fabricate the thin films. (b and d) Powder XRD patterns of the converted 3D FASnI_3_ (red) and CsSnI_3_ (blue) NC thin film from the 2D [R–NH_3_]_2_SnI_4_ RP perovskite, respectively. (c and e) UV–visible spectroscopy of the formation of the 3D FASnI_3_ (red) and CsSnI_3_ (blue) nanocrystal thin film from the 2D [R–NH_3_]_2_SnI_4_ RP structure annealed at 30 °C and 100 °C for 1 h. * marks the peak arising from the glass substrate in the dome XRD sample holder. The XRD references are adapted from ref. 3 for FASnI_3_ (cubic, *Pm*3*m*) and ref. 2 for CsSnI_3_ (orthorhombic, *Pnma*).

We demonstrated that the cation exchange reactions can be successfully performed in solution converting the non-colloidal suspension of 2D [R–NH_3_]_2_SnX_4_ RP nanostructures into 3D ASnX_3_ NCs at room temperature. While this process can have advantages in precisely controlling the synthesis of ASnX_3_ NCs, for most optoelectronic applications, those transformations are desired in thin films.^35,36^ So far, a few reports have successfully demonstrated facile cation exchange reactions on Sn-halide and mixed Pb–Sn halide perovskite bulk thin films, which transforms the dimensionality of the perovskite structure from 2D to 3D.^16,34,37^ However, there are no similar reports showcasing the dimensionality transformation with the existence of a strong confinement effect indicative of newly fabricated NCs. To fabricate high-quality thin films of the non-colloidal suspension, we utilized the modified drop-casting methodology illustrated in Fig. 4(a), which helped in the uniform outspread of the 2D [R–NH_3_]_2_SnX_4_ RP perovskite nanostructures over a glass substrate, taking advantage of the buoyant force.^38–40^ Toluene is used to wet the surface of the substrate for a homogeneous spread, which is then naturally allowed to dry over a period of time (≈20–30 min). When fully dried, we dropped 30 μL of 0.05 M FA(Ol) and 20 μL of 0.05 M Cs(Ol) precursor solutions on the 2D [R–NH_3_]_2_SnX_4_ RP perovskite thin film, which quickly changed its colour from deep red to dark brown as a result of the cation exchange reaction. We also tried the spin-coating procedure but found it to be unsuitable for fabricating a thin film of the non-colloidal suspension.

The morphology of these 2D [R–NH_3_]_2_SnX_4_ RP perovskite thin films before cation exchange can be seen in Fig. S6(a) and (b)† for the bromide and iodide cases, respectively. After naturally drying the 2D [R–NH_3_]_2_SnX_4_ RP perovskite thin film, the FA(Ol)/Cs(Ol) precursor solution (0.05 M) was slowly dropped on it and annealed for an hour at different temperatures (from 30 °C to 100 °C) for the recrystallization process. The converted thin films showed negligible diffraction peaks pertaining to the 2D [R–NH_3_]_2_SnX_4_ RP perovskite 2*θ* values from 1 to 10 (Fig. 4(b) and (d)). The cation exchange with FA^+^ led to a cubic crystal structure (*Pm*3*m*) while with Cs^+^ it led to an orthorhombic crystal phase (*Pnma*) (as measured using a bench-top XRD system with reasonable resolution). This observation is consistent with the crystal phases observed in cation exchange in the non-colloidal suspension (Fig. 2). However, we noticed that in the case of FA^+^, the FASnI_3_ NC thin film had a preferred ordering along the [111] lattice planes. In Fig. 4(c) and (e), we show the absorbance spectra of the obtained thin films recorded in the transmission mode. Both FA^+^ and Cs^+^ systems displayed a similar red shift as observed for solutions with an insignificant effect of the annealing temperature. The excitonic peak corresponding to FASnI_3_ and CsSnI_3_ NCs in thin films was observed at 675 and 696 nm. We used various concentrations (from 0.002 to 0.05 M) and annealing temperatures (from 30 to 100 °C) for the conversion of 2D [R–NH_3_]_2_SnBr_4_ (green) and [R–NH_3_]_2_SnI_4_ (brown) with the FA^+^ cation (Fig. S7†). A higher annealing temperature led to the broadening of the excitonic feature towards the bandgap of the bulk materials, which is consistent with the formation of more bulk in the thin films. For [R–NH_3_]_2_SnX_4_, the increase in the concentration of the Cs^+^ cation led to the degradation of the nanostructures to form a white coloured thin film corresponding cesium halides (Fig. S8†).

### Anion exchange in Sn-halide perovskite nanostructures

We have shown that the cation exchange processes offer a large playground for the Sn-halide perovskite chemistry. Next, we aimed to probe the mobility of anions in our systems. Although anion exchange processes have been demonstrated to be fast and facile in the Pb-halide perovskite NCs, these processes are kinetically driven under a concentration gradient of the exchange anion.^6,17,18,41^ For Sn-halide perovskite NCs, there is no post-synthetic anion exchange report.

**Fig. 5:**
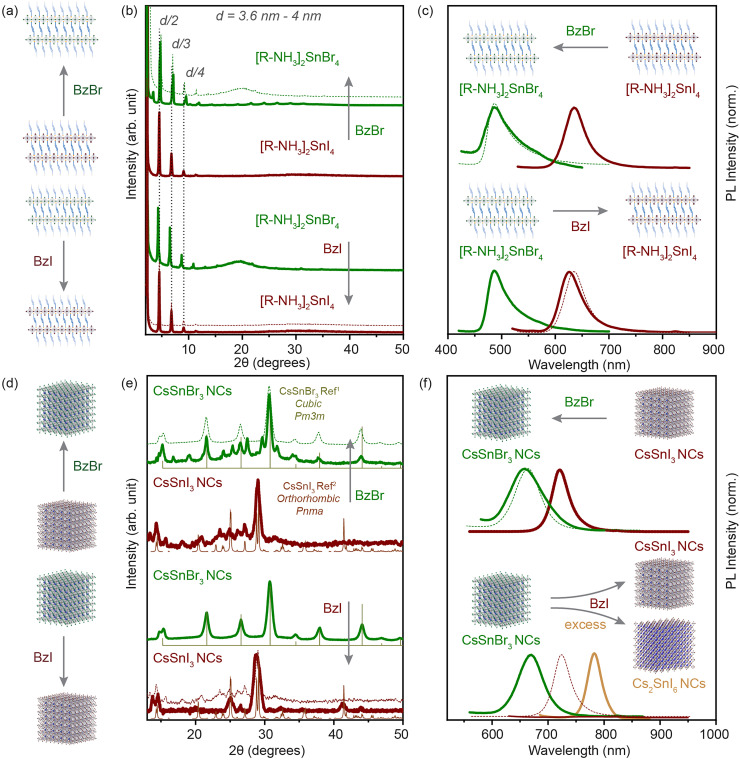
Anion-exchange processes in solution. (a) and (d) illustrate the direction of the anion exchange reaction in the 2D RP nanostructures and the 3D NCs. (b and c) The powder XRD pattern and PL spectra of the 2D RP nanostructures in comparison with the anion-exchanged product for iodide (brown) and bromide (green). (e and f) The XRD pattern and PL spectra of the 3D NCs in comparison with the anion-exchanged product for iodide (brown) and bromide (green). The XRD references are plotted for CsSnBr_3_ cubic *Pm*3*m* (light green) and CsSnI_3_ orthorhombic *Pnma* (light brown).^1,2^ Dotted lines in each plot shows the feature of the directly synthesized product for comparison.

For our systems, we used benzoyl halide as an exchange halide source. In a typical experiment, we loaded a glass vial with 100 μL of 2D RP nanostructures (*C* ≈ 3 mg mL^−1^) or 200 μL of 3D NCs (*C* ≈ 20 mg mL^−1^) and diluted it with 1.5 mL of toluene. To this system, a BzX stock solution (50 to 300 μL) was added with constant stirring, and the mixture was kept for 10 min and then purified as mentioned in the Experimental section. We observed an anion exchange reaction in 2D RP Sn-halide nanostructures and in 3D CsSnX_3_ NCs within a few minutes as illustrated in Fig. 5(a) and (d). The XRD pattern shown in Fig. 5(b) confirmed the presence of 2D RP nanostructures after the anion exchange for each halide, with the lattice spacing varying from 3.6 to 4 nm depending on the extent of the nanostructures towards the [100] plane in the exchanged perovskite. The changes in the *d*-spacing were relatively smaller after the halide exchange for the exchanged structure (≈4 times the difference between the Sn–X bond lengths). Thus, the XRD pattern only confirms the presence of 2D RP nanostructures after the anion-exchange. The steady-state PL of the directly synthesized 2D RP nanostructures as can be observed from Fig. 5(c) shows a peak at 488 nm for [R–NH_3_]_2_SnBr_4_ and at 635 nm for [R–NH_3_]_2_SnI_4_. After the bromide exchange, the PL peak of the exchanged 2D [R–NH_3_]_2_SnBr_4_ was blue shifted to 488 nm, presenting a similar band gap to the directly synthesized (no anion exchange) 2D [R–NH_3_]_2_SnBr_4_. Similarly, for the iodide exchange, the PL peak of the exchanged 2D [R–NH_3_]_2_SnI_4_ was red shifted to 630 nm. The restoration of the PL peak to the directly synthesized 2D RP nanostructures confirms the anion exchange reaction.

We applied a similar approach to the 3D CsSnX_3_ NCs. Fig. 5(e) shows the XRD pattern for the directly synthesized 3D NCs in comparison with the anion-exchanged NCs (experimental details in the Experimental section). The iodide exchanged CsSnI_3_ NCs show full transformation into an orthorhombic *Pnma* crystal structure from apparently cubic *Pm*3*m* CsSnBr_3_ NCs. However, the well-defined PL peak disappeared after the anion exchange process most probably due to low PLQY, a result of self p-doping, which is a result of oxidation of the Sn^2+^ cation especially on the surface of NCs (Fig. 5(f)) as shown also in previous studies.^42^ Note that if the BzI used for the iodide exchange was added in an excessive amount or it was synthesized for more than a week in advance, the obtained material would be correlated to the XRD pattern of Cs_2_SnI_6_ in a cubic *Fm*3*m* (Fig. S11†). This observation was in good agreement with the PL spectroscopy where we identified a shift of the initial peak to 780 nm (Fig. 5(f)). Fig. S12† shows the evolution of the crystal structure from CsSnI_3_ (orthorhombic, *Pnma*) to Cs_2_SnI_6_ (cubic, *Fm*3*m*) when an increasing amount of BzI was added. The bromide exchanged CsSnI_3_ NCs showed the transformation into the cubic *Pm*3*m* crystal phase majorly with the negligible presence of the CsSnI_3_ orthorhombic *Pnma* diffraction peaks, which does not indicate a complete halide exchange process. (Note that we have associated the crystal structure after analyzing bench-top XRD measurements in a protected environment.) However, the PL peak blue shifted to 665 nm, which correlated well with the directly synthesized CsSnBr_3_ NCs. In addition, the increase in FWHM (≈0.02 eV) indicated the increase in size-dispersity of the exchanged NCs. We further probed the anion exchange from CsSnBr_3_ NCs to CsSnCl_3_ NCs *via* BzCl. The blue shift was observed in the absorbance and PL spectra after the addition of BzCl, however, due to the instability of the crystal structure, we were unable to perform any structural characterization (Fig. S9–S11†).

## Conclusions

The ion exchange processes within Sn-halide perovskite nanostructures facilitated facile compositional tuning, inducing systematic alterations in band gap characteristics through either the modulation of dimensionality, as observed in cation exchange, or the modification of the crystal structure, as evidenced in anion exchange. Notably, cation exchange transitioned seamlessly, resulting in the transformation of two-dimensional [R–NH_3_]_2_SnX_4_ RP perovskite nanostructures into three-dimensional nanocrystals (3D NCs) at room temperature, which is particularly evident in the iodide counterparts in comparison with bromide. The thin films composed of 2D [R–NH_3_]_2_SnX_4_ RP perovskite nanostructures exhibited diffusion of smaller cations through a liquid–solid interface, culminating in the conversion to higher-dimensional 3D NCs. Anion exchange processes, utilizing benzoyl halide sources, were conducted on both 2D [R–NH_3_]_2_SnX_4_ RP nanostructures and 3D NCs. The anion exchange to bromide-based 3D NCs induced a crystalline structural transition from orthorhombic to cubic, while the iodide exchange manifested the reverse transformation. This study exemplifies the ion-exchange capabilities inherent in Sn-halide perovskite nanostructures, demonstrating their capacity to traverse inter-dimensional and inter-crystallographic phases under controlled physical and chemical parameters.

## Experimental section

### Materials

Cesium carbonate (Cs_2_CO_3_, 99.9%), formamidine acetate (FAAc, 99%), tin(ii) iodide (anhydrous beads, 99.99%), benzoyl bromide (BzBr, 97%), benzoyl chloride (BzCl, 99%), sodium iodide (NaI, 99.999%), octadecene (ODE, Tech. grade, 90%), oleic acid (OA, tech. grade, 90%) and oleyl amine (OLA, tech. grade, 80–90%) were purchased from Sigma Aldrich. Tin(ii) bromide (SnBr_2_, 99.9%) was purchased from TCI Europe. Toluene (extra dry, 99.85%) was purchased from ACROS organics.

### Synthesis of the formamidium oleate precursor (0.25 M)

Formamidinium acetate (0.26 g, 2.5 mmol), OlAc (1.25 ml, 4 mmol), and ODE (10 ml) were loaded into a 25 ml 3-neck round-bottom flask and vigorously stirred under vacuum for 1 h at 50 °C and then the temperature was increased slowly to 105 °C. The reaction mixture was degassed for 1 h. Now under an N_2_ flow, the reaction mixture was heated up to 160 °C to assure the complete conversion of formamidine acetate salt into formamidinium oleate. The obtained formamidinium oleate solution was carefully transferred to the glove box when cooled to room temperature. This solution was diluted with toluene to obtain a 0.05 M FA(Ol) precursor solution.

### Synthesis of cesium oleate precursor (0.22 M)

Cesium carbonate (0.408 g, 1.25 mmol), OlAc (1.25 ml, 4 mmol), and ODE (10 ml) were loaded into a 25 ml 3-neck round-bottom flask and vigorously stirred under vacuum for 1 h at 120 °C. Furthermore, the reaction mixture was heated up to 180 °C under an N_2_ flow to ensure the complete conversion of cesium carbonate into cesium oleate. The obtained cesium oleate solution was carefully transferred to the glove box when cooled to room temperature. This solution was diluted with toluene to obtain a 0.05 M Cs(Ol) precursor solution and heated to produce a clear solution before the usage.

### Synthesis of the 2D [R–NH_3_]_2_SnX_4_ RP perovskite nanostructures

SnI_2_/SnBr_2_ (2 mmol) along with dried OA (0.63 mL, 2 mmol), OLA (0.66 mL, 2 mmol), and ODE (5 mL) were loaded into a 25 mL 3-neck flask inside a N_2_ glovebox. The reaction mixture was carefully transferred to a Schlenk line and stirred under vacuum for 10 min at room temperature, after which the temperature was increased to 105 °C and the mixture vigorously stirred for 45 min. The temperature was further increased to 200 °C under an N_2_ flow, and the reaction was then cooled to room temperature using an ice-water bath after 5 s. Note: Avoid the shaking of the round-bottom flask at this point as the reaction mixture quickly crystallizes at the cooler surfaces of the flask. The reaction mixture turns dark red in the case of iodide and yellow in the case of bromide. The reaction flask was carefully transferred to the glovebox for the purification step. The crude solution was equally divided into two centrifuge tubes and centrifuged at 5000 rpm (7082 rcf) for 3 min. The clear supernatant was discarded, and the precipitate was redispersed in toluene (5 mL) followed by centrifugation at 13 000 rpm (18 412 rcf) for 5 min. The supernatant was discarded again and the colloidally unstable yellow precipitate was stored in 5 mL of toluene in the glovebox for further experiments.

### Cation exchange of the 2D [R–NH_3_]_2_SnX_4_ RP perovskite nanostructure suspension

The stock solution of the 2D [R–NH_3_]_2_SnX_4_ RP perovskite nanostructures was prepared in toluene with a concentration of ≈3 mg mL^−1^. A magnetic stirrer was placed in a 4 mL glass vial containing 200 μL of the stock solution with 1.5 mL of toluene, and this vial was then injected with the required volumes of 0.05 M FA(Ol)/Cs(Ol) under vigorous stirring to observe the changes in the UV–visible spectra. For a complete cation exchange, 240 μL of 0.05 M FA(Ol) and 120 μL of 0.05 M Cs(Ol) precursor solution injection followed by 30 min of stirring was utilized. For the concentration dependence study, different volumes of the 0.05 M FA(Ol)/Cs(Ol) precursor solution were employed.

### Cation exchange of the 2D [R–NH_3_]_2_SnX_4_ RP perovskite nanostructure thin film

The soda-lime glass substrates (10 mm × 10 mm) were cleaned with soap water and washed using distilled water. The substrates were then subjected to sonication for 15 min in ethanol, dried, and sonicated for another 15 min in acetone, followed by drying with a strong airflow. The cleaned substrates were transferred to the glove box. For the thin-film fabrication of 2D [R–NH_3_]_2_SnX_4_ RP perovskite nanostructures, 20 μL of anhydrous toluene was used to wet the surface of the substrate. Then 3 mg mL^−1^ solution of 2D [R–NH_3_]_2_SnX_4_ was dropcast with the assistance of toluene. This thin film was then dried naturally within 20–30 min. After the film was dried, 30 μL of a 0.05 M FA(Ol) or 20 μL Cs(Ol) solution was dropped on the thin film, which made the appearance of the film darker and more homogeneous. This thin film was then kept for annealing for 1 h at different temperatures to provide time for cation diffusion through liquid–solid interface. After 1 h, the thin film can be washed with the help of 20 μL of anhydrous toluene over a spin-coater.

### Synthesis of the 3D CsSnX_3_ perovskite NCs

The 3D CsSnX_3_ NCs were synthesized utilizing the protocol from Gahlot *et al.*^21^ SnI_2_/SnBr_2_ (2 mmol) along with dried OA (0.63 mL, 2 mmol), OLA (0.66 mL, 2 mmol), and ODE (5 mL) were loaded into a 25 mL 3-neck flask inside a N_2_ glovebox. The reaction mixture was carefully transferred to a Schlenk line and stirred under vacuum for 10 min at room temperature, after which the temperature was increased to 105 °C and the mixture vigorously stirred for 45 min. The temperature was further increased to 200 °C under an N_2_ flow, and 2.8 mL (0.622 mmol) of 0.222 M cesium oleate was swiftly injected. The reaction was stopped after 25 s by quickly immersing the reaction flask in an ice-water bath. The reaction flask was carefully transferred to the glovebox for the purification step. The crude solution was equally divided into two centrifuge tubes and centrifuged at 5000 rpm (7082 rcf) for 3 min. The supernatant was discarded, and the precipitate was redispersed in toluene (5 mL) followed by centrifugation at 13 000 rpm (18 412 rcf) for 5 min. The supernatant was discarded and the precipitate was re-dispersed in 5 mL of toluene, and stored in the glovebox for further experiments.

### Synthesis of benzoyl iodide (BzI)

Benzoyl iodide was synthesized following the modified procedure from Theobald and Smith.^43^ In the glove box, benzoyl chloride (2.8 mL) was added to a 20 mL glass vial containing 6 g of sodium iodide. This reaction mixture was vigorously stirred overnight at 80 °C for 6 h. The dark orange-red solution was diluted with 3 mL toluene and filtered with a 0.45 μm PTFE filter. This solution was then utilized for anion exchange in 2D [R–NH_3_]_2_SnX_4_ RP perovskite nanostructures and 3D CsSnX_3_ perovskite NCs. It would be best to utilize the fresh BzI solution.

### Anion exchange of the 2D [R–NH_3_]_2_SnX_4_ RP perovskite nanostructures

The stock solution of 2D [R–NH_3_]_2_SnX_4_ RP perovskite nanostructures was prepared in toluene with a concentration of ≈3 mg mL^−1^. 100 μL of the stock solution was mixed with 1.5 mL of toluene, 100 μL of BzI was added to 2D [R–NH_3_]_2_SnBr_4_/100 μL of BzBr (v/v – 1/9 in toluene) was added to 2D [R–NH_3_]_2_SnI_4_ for an anion exchange reaction. The reaction was stirred for 10 min. After 10 min, the reaction mixture was transferred to a centrifuge tube. The reaction mixture was centrifuged at 13 000 rpm (18 412 rcf) for 5 min, the supernatant was discarded, and the precipitate was redissolved in 3 ml of toluene, which was again centrifuged using the previous parameters. More supernatant was discarded, and the precipitate was redissolved in 3 ml of toluene and utilized for further characterization.

### Anion exchange of CsSnI_3_ perovskite NCs

A stock solution of 20 mg mL^−1^ of freshly prepared CsSnI_3_ NCs in toluene was prepared. The BzBr and BzCl precursor solutions were prepared by dissolving 100 μL in 5 mL toluene. For an anion exchange experiment, 200 μL of CsSnI_3_ NCs was mixed with 1.5 mL of toluene in a 4 mL glass vial with a magnetic stirrer. 300 μL of the BzBr solution was added and stirred for 1 h. The reaction mixture was then centrifuged at 13 000 rpm (18 412 rcf) for 5 min. The supernatant was discarded and the precipitate was redissolved in 1.5 mL of toluene. Then for a Cl exchange, the same solution, added with 300 μL of BzCl, was stirred for 1 h. The reaction mixture was then centrifuged at 13 000 rpm (18 412 rcf) for 5 min. The supernatant was discarded and the precipitate was redissolved in 1.5 mL of toluene. For further structural and optical characterization, the precipitate obtained was re-dissolved in 2 ml of toluene and the solution was centrifuged at 2000 rpm for 3 min. The precipitate was discarded and the supernatant was used. These anion exchange processes are air and time-sensitive in nature.

### Anion exchange of CsSnBr_3_ perovskite NCs

A stock solution of 20 mg mL^−1^ CsSnBr_3_ NCs in toluene was prepared. The solutions of freshly prepared BzI and BzCl were used in the same concentration mentioned above. For an anion exchange experiment, 200 μL of CsSnBr_3_ NCs was mixed with 1.5 mL of toluene in a 4 mL glass vial with a magnetic stirrer. 50 μL of the BzI solution and 100 μL of the BzCl solution were added and the mixture was stirred for 1 h to obtain CsSnI_3_ and CsSnCl_3_ NCs, respectively. The reaction mixture was then centrifuged at 13 000 rpm (18 412 rcf) for 5 min. The supernatant was discarded and the precipitate was redissolved in 1.5 mL of toluene. The precipitate obtained was re-dissolved in 2 ml of toluene and centrifuged at 2000 rpm for 3 min. The precipitate was discarded and the supernatant was used for characterization. These anion exchange processes are air and time-sensitive in nature.

### Characterization

All measurements were performed in an air-free environment. Powder X-ray diffraction measurements were performed in an inert dome sample holder in a Bruker D8 Advanced diffractometer with Bragg–Brentano geometry using Cu Kα radiation (*λ* = 1.54 Å) and a Lynxeye detector. SEM images were acquired *via* a FEI Helios G4 CX electron microscope in the transmission mode operated at 18 kV with 1.4 nAmp beam current using an ultrathin grid with 400 mesh, Cu (Ted Pella, Inc. 01822-F) for NCs while the lens detector mode (TLD) was used for thin films. The absorption measurements were performed on a table-top Avantes UV–vis spectrophotometer using a tungsten and halogen filament lamp as the excitation source. The steady-state photoluminescence measurements were performed on a Horiba Scientific Jobin Yvon spectrometer equipped with a PMT detector.

## Conflicts of interest

The authors declare no conflict of interest.

## Supplementary Material

NR-016-D3NR06075F-s001
