# Supplementary material for: Structural and optical control through anion and cation exchange processes for Sn-halide perovskite nanostructures
Source: Nanoscale. 2024 Feb 6;16(10):5177–87. doi: 10.1039/d3nr06075f (PMC10918525; doi:10.1039/d3nr06075f)
Supplement: NR-016-D3NR06075F-s001 [file NR-016-D3NR06075F-s001.pdf]

Electronic Supplementary Material (ESI) for Nanoscale.
This journal is © The Royal Society of Chemistry 2024

Supporting Information for

Structural and Optical Control through Anion and Cation Exchange Processes for Sn-Halide Perovskite Nanostructures

Kushagra Gahlot,[1] *Julius Meijer*[1] *and Loredana Protesescu*[1,]*

Zernike Institute for Advanced Materials, University of Groningen, Nijenborgh 4, Groningen, 9747AG, The Netherlands.

Corresponding author: Dr. Loredana Protesescu, E-mail: l.protesescu@rug.nl

Contents

	Description	Page
**Figure S1**	Powder X-ray diffraction pattern of 2D RP structures quenched with and without ice-water.	3
**Figure S2**	Powder X-ray diffraction pattern and UV-Visible spectroscopy before and after cation exchange in 2D $[R\text{-}NH_3]_2SnBr_4$ RP perovskite nanostructures.	4
**Figure S3**	SEM images (transmission mode at 18 kV) of $FASnI_3$ NCs formed via cation exchange of 2D $[R\text{-}NH_3]_2SnI_4$ RP nanostructures.	5
**Figure S4**	Extended time-dependent UV-Visible spectroscopy measurements of the cation exchange reaction.	6
**Figure S5**	Concentration dependent UV-Visible spectroscopy measurements of cation exchange in 2D $[R\text{-}NH_3]_2SnBr_4$ RP perovskite nanostructures.	7
**Figure S6**	SEM images (transmission mode at 18 kV) of 2D $[R\text{-}NH_3]_2SnX_4$ RP perovskite nanostructures in lens detector mode.	8
**Figure S7**	Cation Exchange in thin-films - Annealing temperature dependence in Sn-halide perovskite thin-films with A cation addition at different concentrations.	9
**Figure S8**	X-ray diffraction pattern of degraded thin-film after excess Cs(Ol) addition for cation exchange.	10
**Figure S9**	UV-Visible and PL spectroscopy of anion exchange on $CsSnI_3$ NCs.	10
**Figure S10**	Anion exchange process via SEM Micrographs from $CsSnI_3$ NCs to $CsSnBr_3$ NCs and $CsSnBr_3$ NCs to $CsSnCl_3$ NCs.	11
**Figure S11**	Powder X-ray diffraction pattern, UV-Visible and PL spectroscopy of anion exchange on $CsSnBr_3$ NCs.	11
**Figure S12**	X-ray diffraction pattern evolution with different amount of BzI added for anion exchange	12

References 12

Supporting Figures

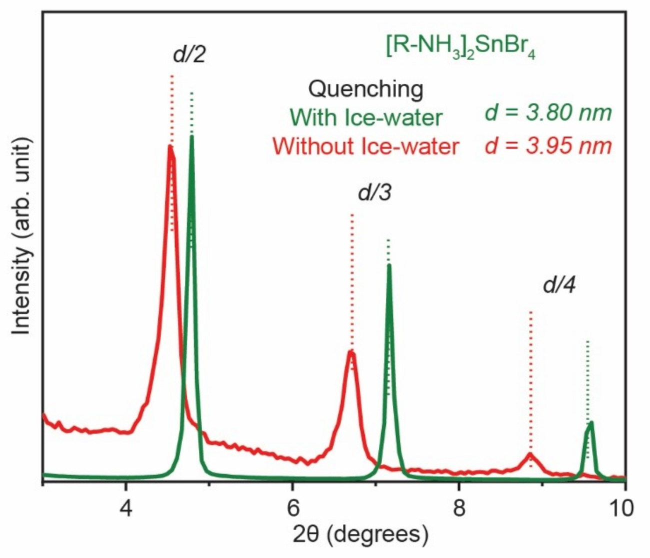

Figure S1. Powder X-ray diffraction pattern of the 2D [R-NH$_3$]$_2$SnBr$_4$ n = 1 RP nanostructures with reaction quenched with ice-water (green) and without ice-water (red).

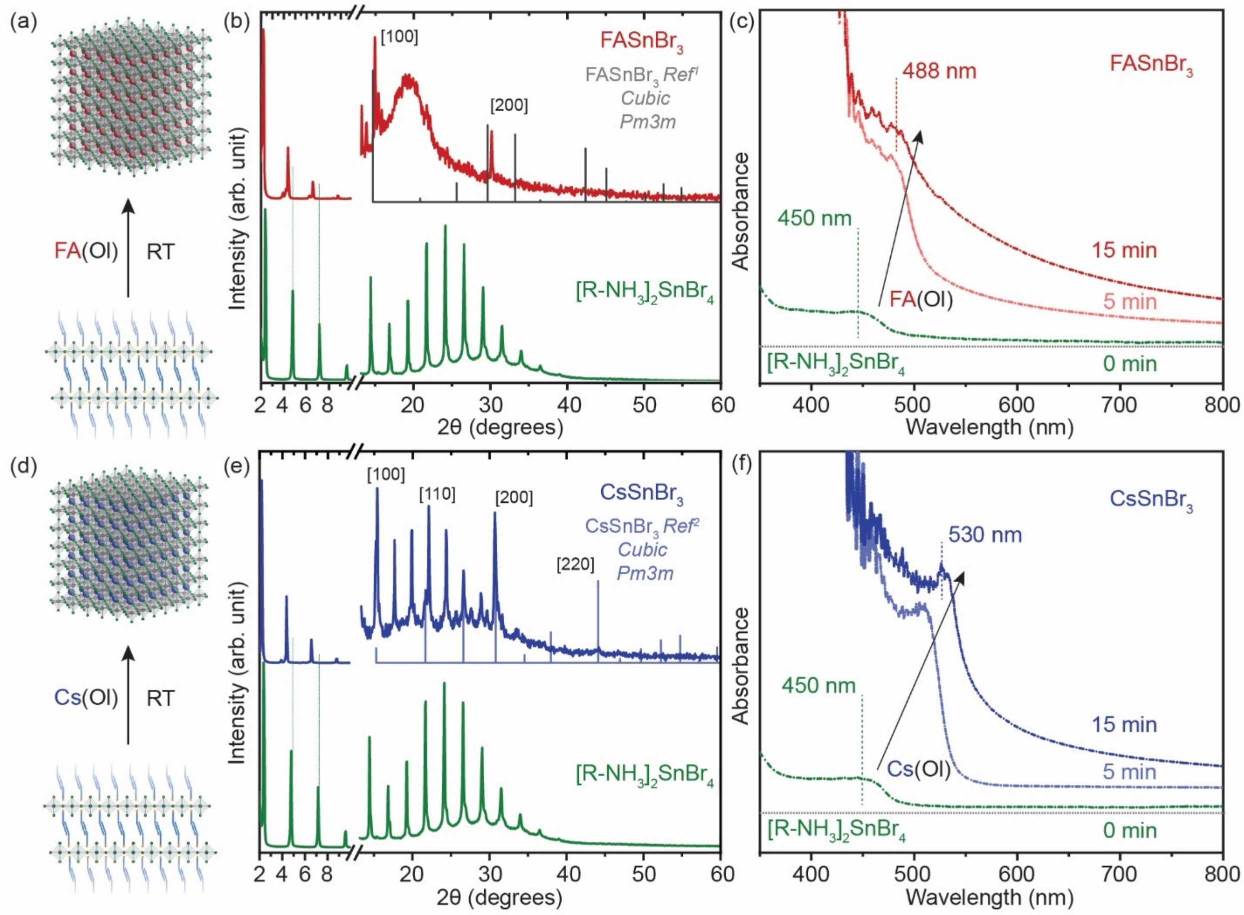

Figure S2. (a) and (d) Schematic representation of conversion of 2D [R-NH_3]$_2$$SnBr_4$ to 3D $FASnBr_3$ and $CsSnBr_3$ NCs respectively. (b) and (e) Powder X-ray diffraction pattern of the converted 3D $FASnBr_3$ (red) and $CsSnBr_3$ (blue) NCs from 2D [R-NH_3]$_2$$SnBr_4$ respectively. (c) and (f) In-situ UV-Visible spectroscopy of the formation of 3D $FASnBr_3$ (red) and $CsSnBr_3$ (blue) NCs from 2D [R-NH_3]$_2$$SnBr_4$ RP structure with the addition of A cation. The XRD references were adapted from ref.[1] for $FASnBr_3$ (cubic, Pm3m) and ref.[2] for $CsSnBr_3$ (cubic, Pm3m).

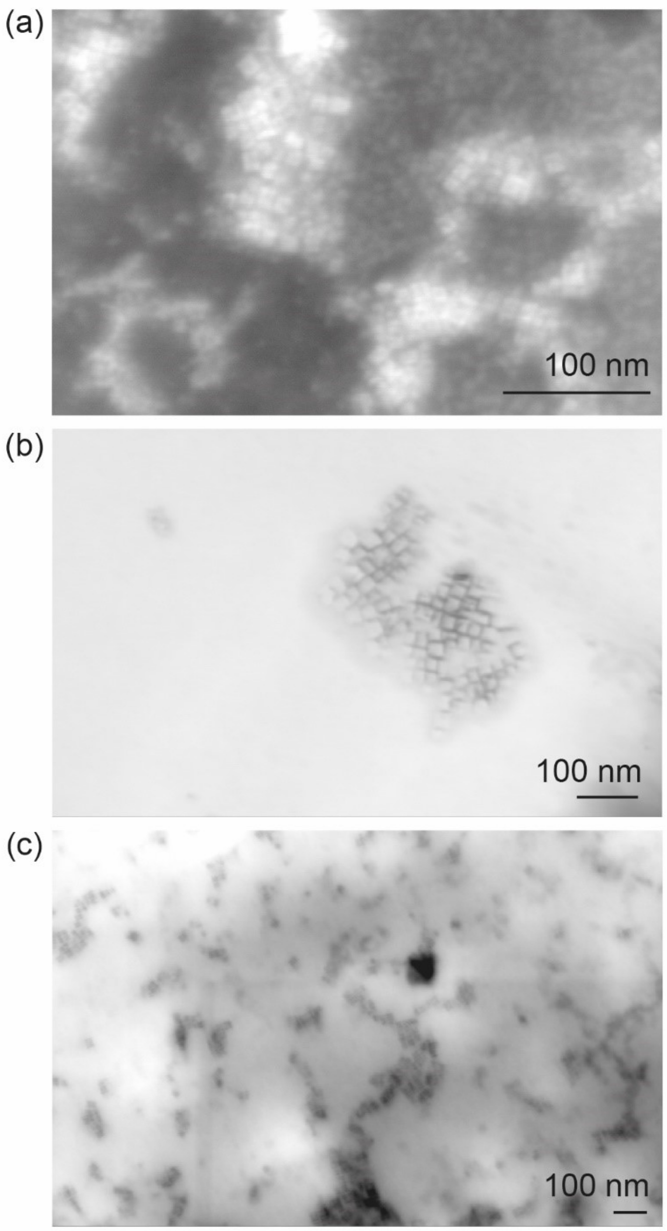

Figure S3. SEM images of $FASnI_3$ NCs formed via cation exchange of 2D $[R\text{-}NH_3]_2SnI_4$ RP nanostructures at decreasing magnifications from (a) to (c).

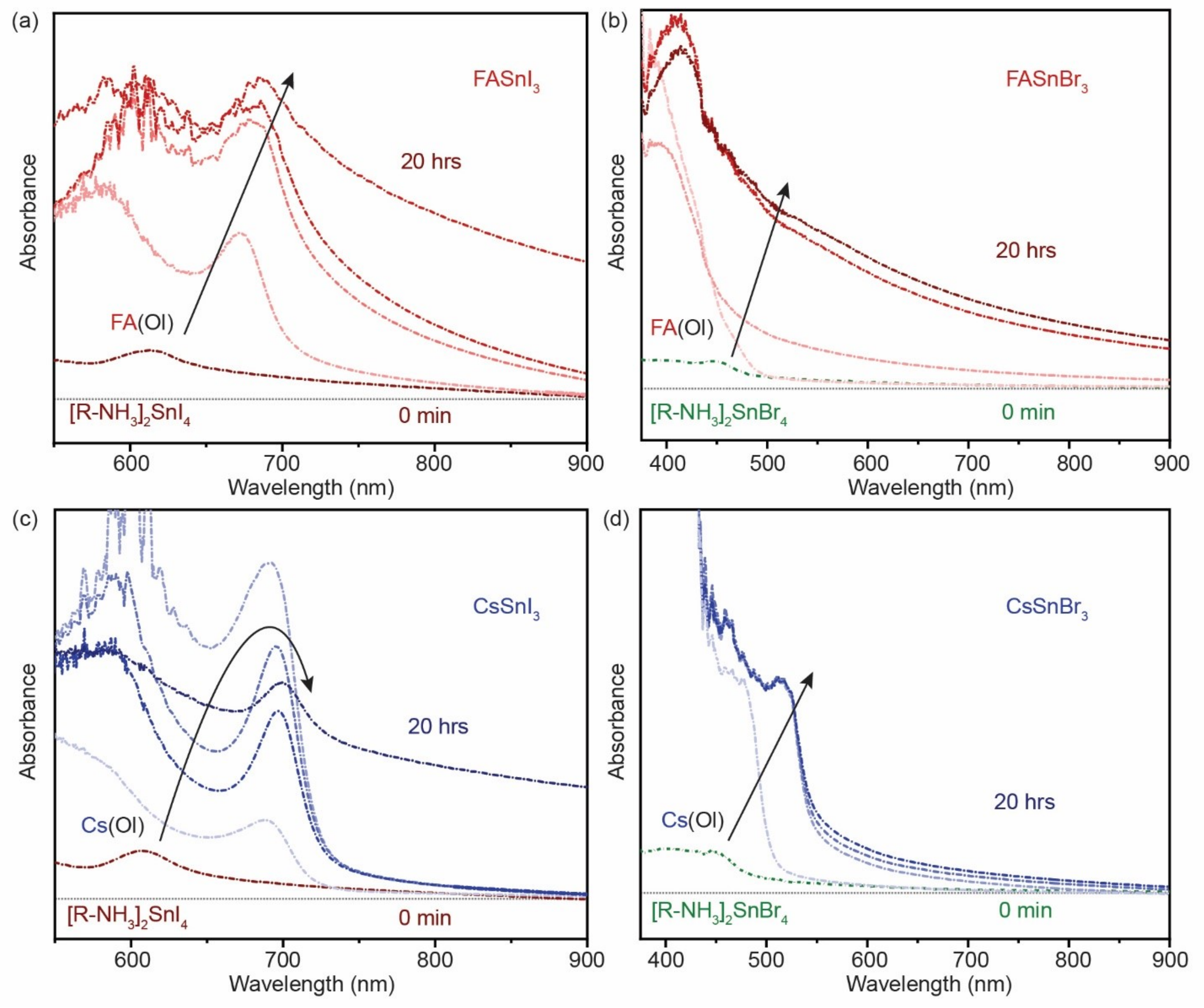

Figure S4. Extended time-dependence optical measurements (a) and (b) Insitu UV-Visible spectroscopy of the formation of 3D $FASnI_3$ and $FASnBr_3$ NCswith the addition of FA(Ol) in 2D $[R\text{-}NH_3]_2SnI_4$ and $[R\text{-}NH_3]_2SnBr_4$ RP structures respectively over the time of 30 minutes. (c) and (d) Insitu UV-Visible spectroscopy of the formation of 3D $CsSnI_3$ and $CsSnBr_3$ NCswith the addition of Cs(Ol) in 2D $[R\text{-}NH_3]_2SnI_4$ and $[R\text{-}NH_3]_2SnBr_4$ RP structures respectively over the time of 30 minutes.

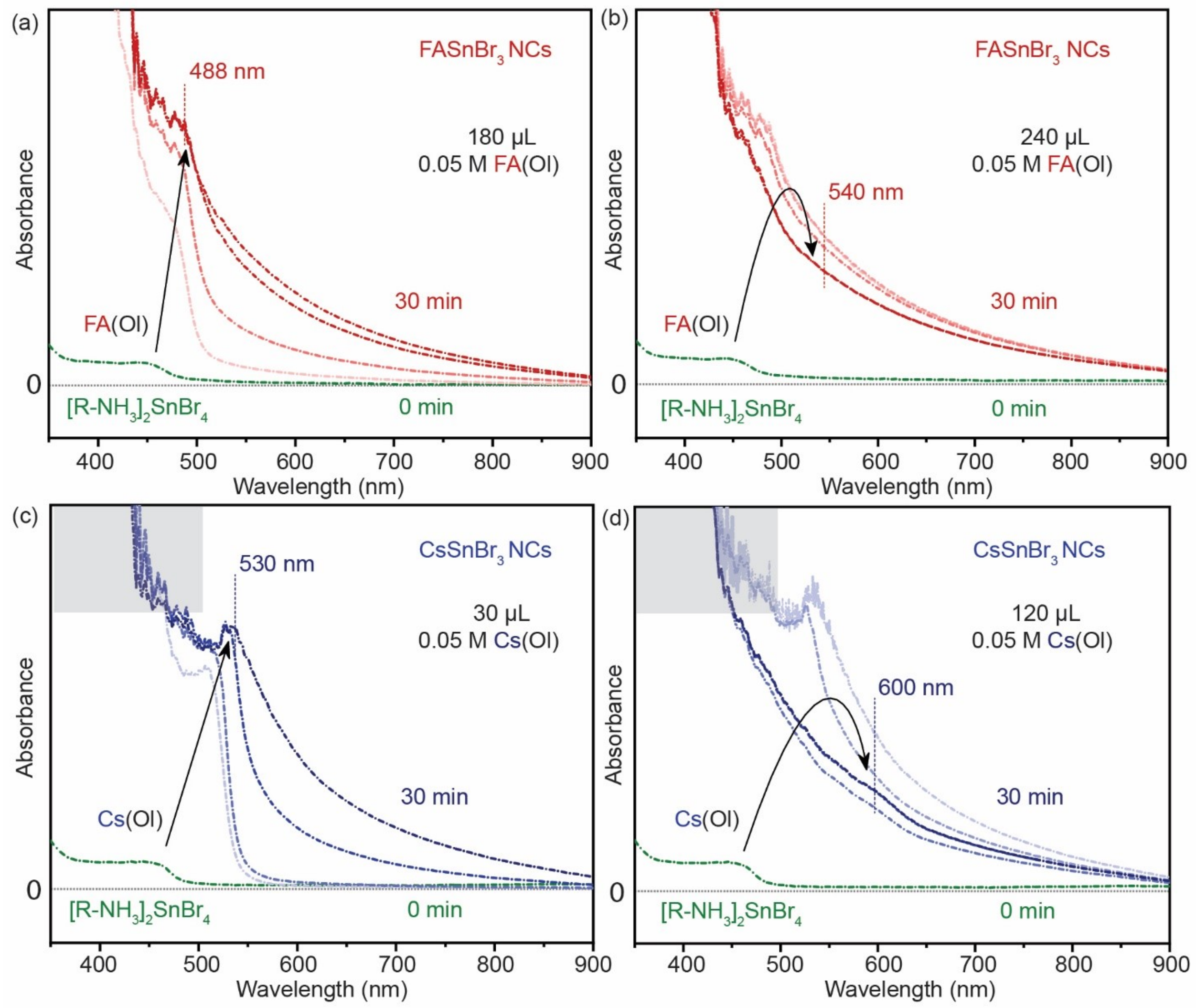

Figure S5. Concentration dependence (a) and (b) Insitu UV-Visible spectroscopy of the formation of 3D $FASnBr_3$ (red) NCswith the addition of FA(Ol) in 2D $[R\text{-}NH_3]_2SnBr_4$ RP structures over the time of 30 minutes. (c) and (d) Insitu UV-Visible spectroscopy of the formation of 3D $CsSnBr_3$ (blue) NCswith the addition of Cs(Ol) in 2D $[R\text{-}NH_3]_2SnBr_4$ RP structures over the time of 30 minutes.

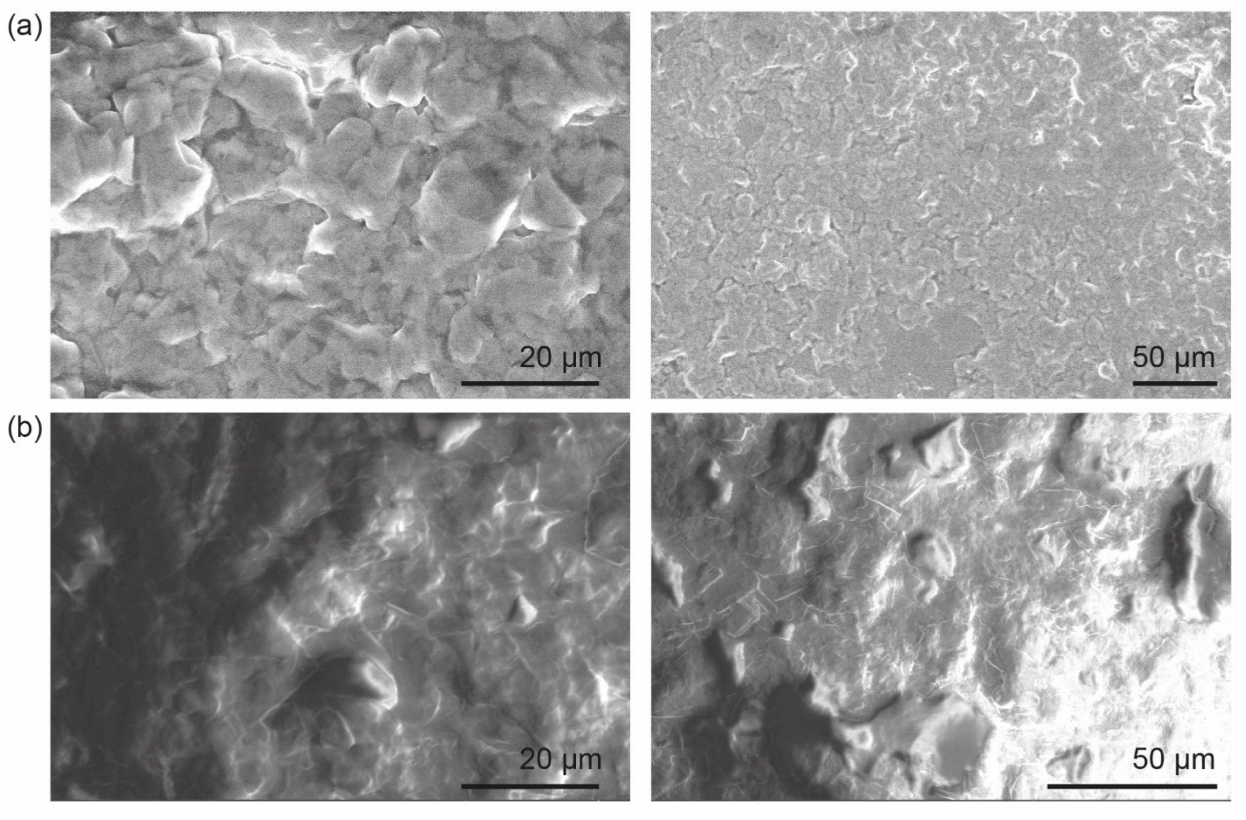

Figure S6. SEM images in the through the lens (TLD) mode. (a) Thin-film of 2D $[R\text{-}NH_3]_2SnBr_4$ RP nanostructures. (b) Thin-film of 2D $[R\text{-}NH_3]_2SnI_4$ RP nanostructures.

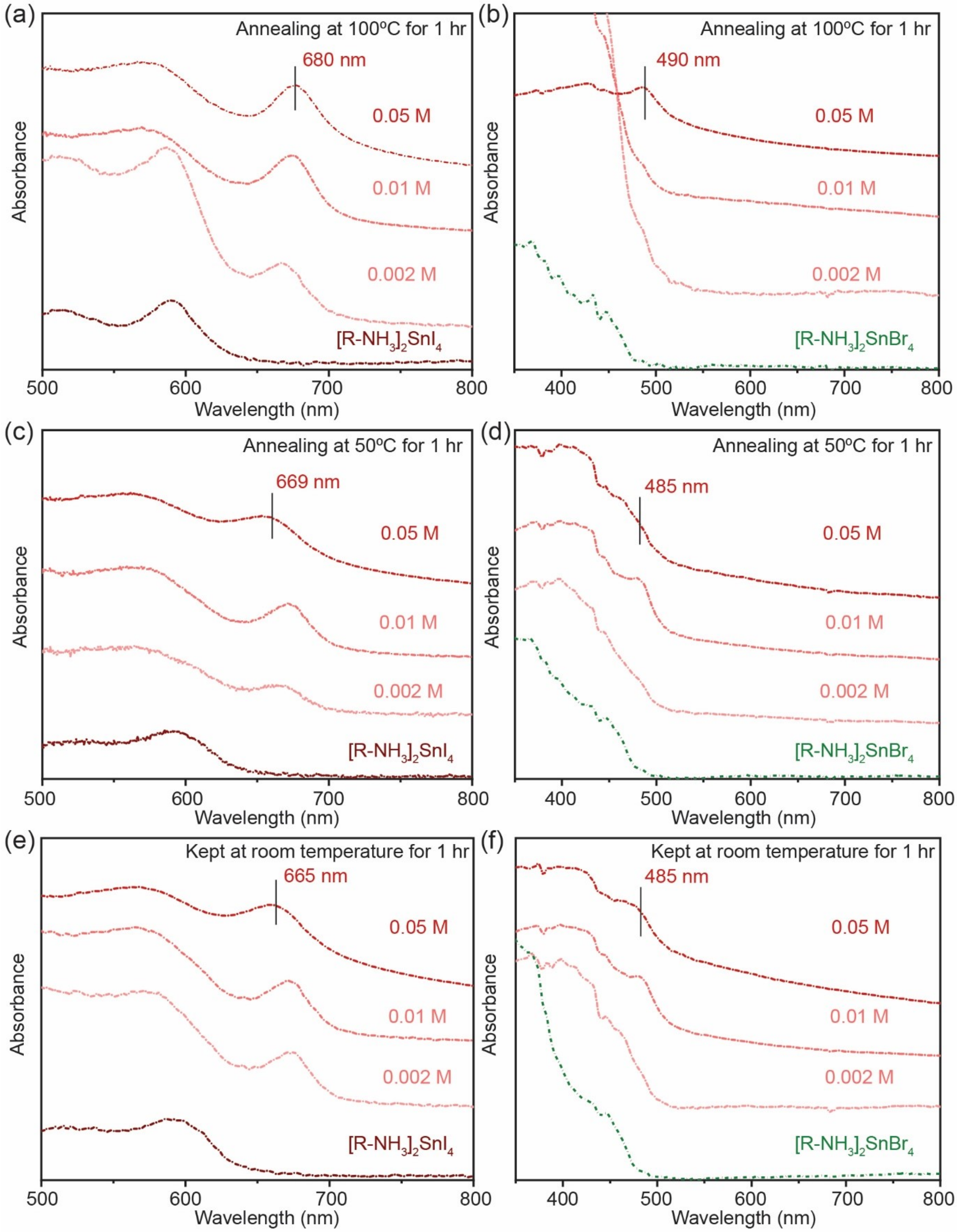

Figure S7. Annealing temperature dependence in Sn-halide perovskite thin-films with A cation addition at different concentrations. UV-Visible spectroscopy of the formation of 3D $FASnI_3$ and 3D $FASnBr_3$ nanostructures with the addition of FA(Ol) on thin-film of 2D $[R\text{-}NH_3]_2SnI_4$ RP nanostructures annealed at room temperature ((e) and (f)), 50ºC ((c) and (d)) and 100ºC ((a) and (b)) for an hour.

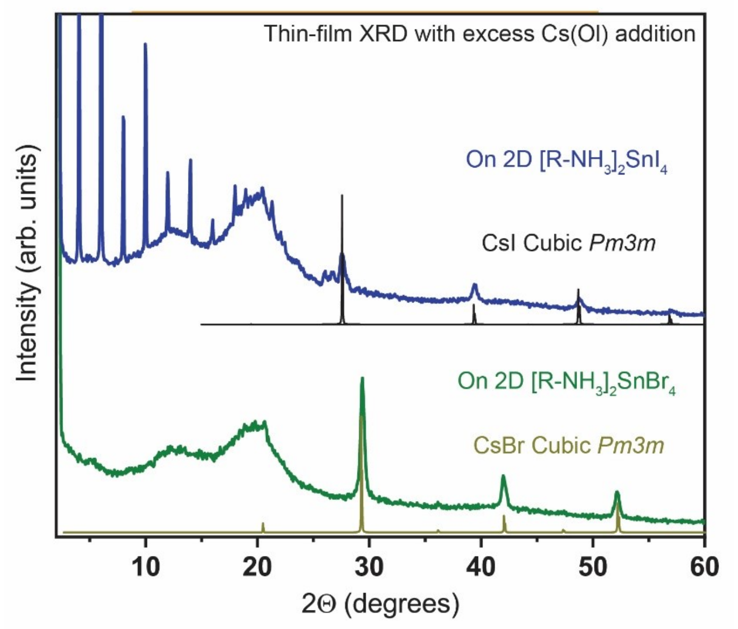

Figure S8. Powder X-ray diffraction pattern of the degraded thin-film with excess addition of Cs(Ol) solution (30 µL of 0.05 M solution) on 2D $[R\text{-}NH_3]_2SnBr_4$ (green) and 2D $[R\text{-}NH_3]_2SnI_4$ (blue). .The XRD bulk references are plotted for CsI (cubic, Pm3m) and CsBr (cubic, Pm3m).[3]

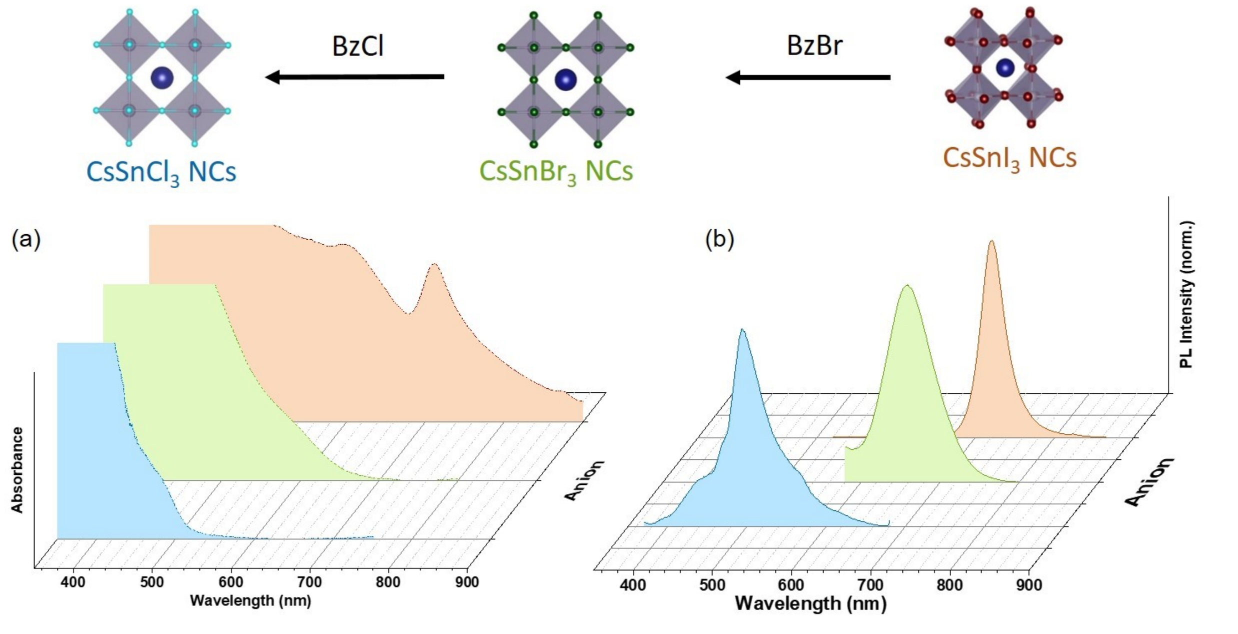

Figure S9. Anion exchange processes in solution of $CsSnI_3$ NCs. (a) UV-Visible spectroscopy of 3D $CsSnBr_3$ NCs formed via Br anion exchange of 3D $CsSnI_3$ NCs performed at room temperature which further exchanged with Cl to form 3D $CsSnCl_3$ NCs. (b) Photoluminescence spectroscopy of 3D $CsSnBr_3$ NCs formed via Br anion exchange of 3D $CsSnI_3$ NCs performed at room temperature which further exchanged with Cl to form 3D $CsSnCl_3$ NCs.

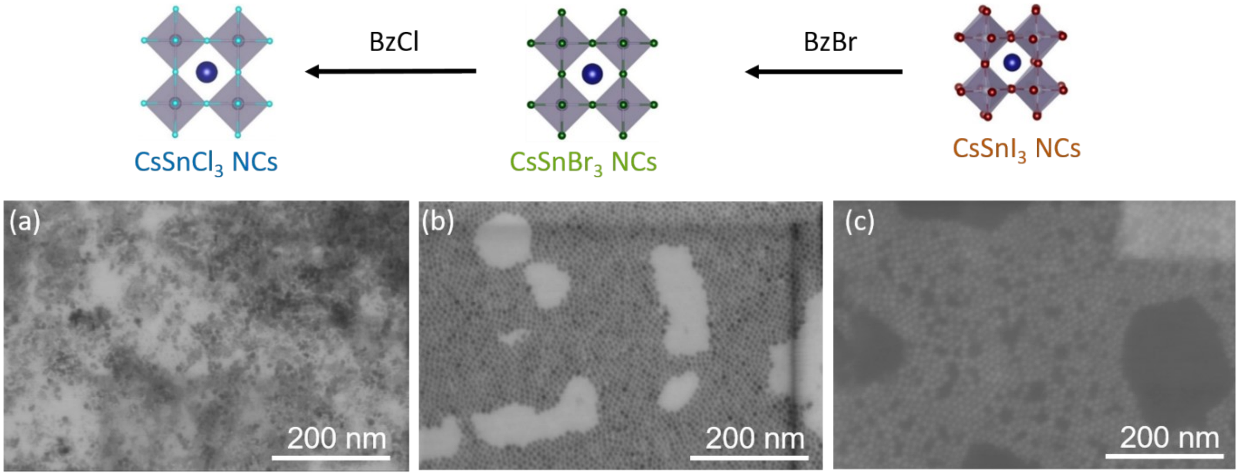

Figure S10. Anion exchange processes in solution of $CsSnI_3$ NCs. (a) and (b) show the SEM micrographs of anion-exchanged 3D $CsSnBr_3$ NCs formed via Br exchange of 3D $CsSnI_3$ NCs and 3D $CsSnCl_3$ NCs via a Cl exchange of 3D $CsSnBr_3$ NCs formed earlier respectively. (c) SEM micrographs of $CsSnI_3$ NCs before anion exchange.

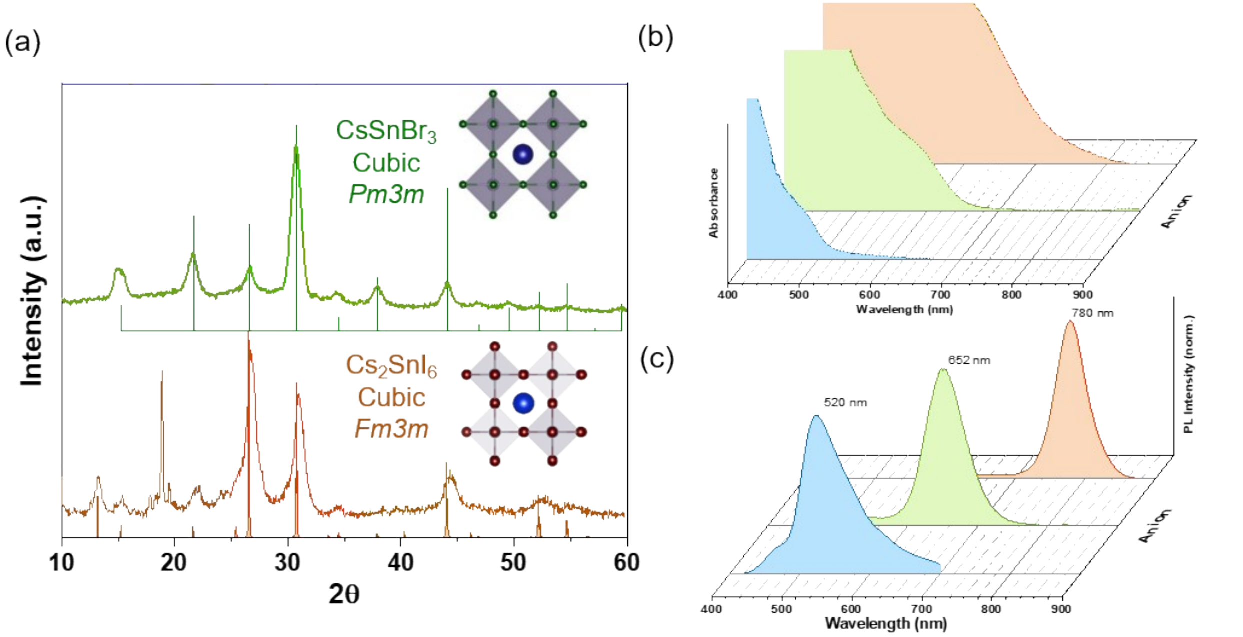

Figure S11. Anion exchange processes in solution of $CsSnBr_3$ NCs. (a) Powder X-ray diffraction pattern of the Iodide exchanged $CsSnBr_3$ NCs which yield Cs_2SnI_6 NCs with excess BzI addition. (b) and (c) UV-Visible and PL spectroscopy anion exchanged sample of $CsSnCl_3$ *NCs (blue) and Cs_2SnI_6 NCs (dark orange) with starting composition $CsSnBr_3$ NCs for comparison. The XRD bulk references are plotted from $CsSnBr_3$ (cubic, Pm3m)[2] and Cs_2SnI_6 (cubic, Fm3m)[4]*

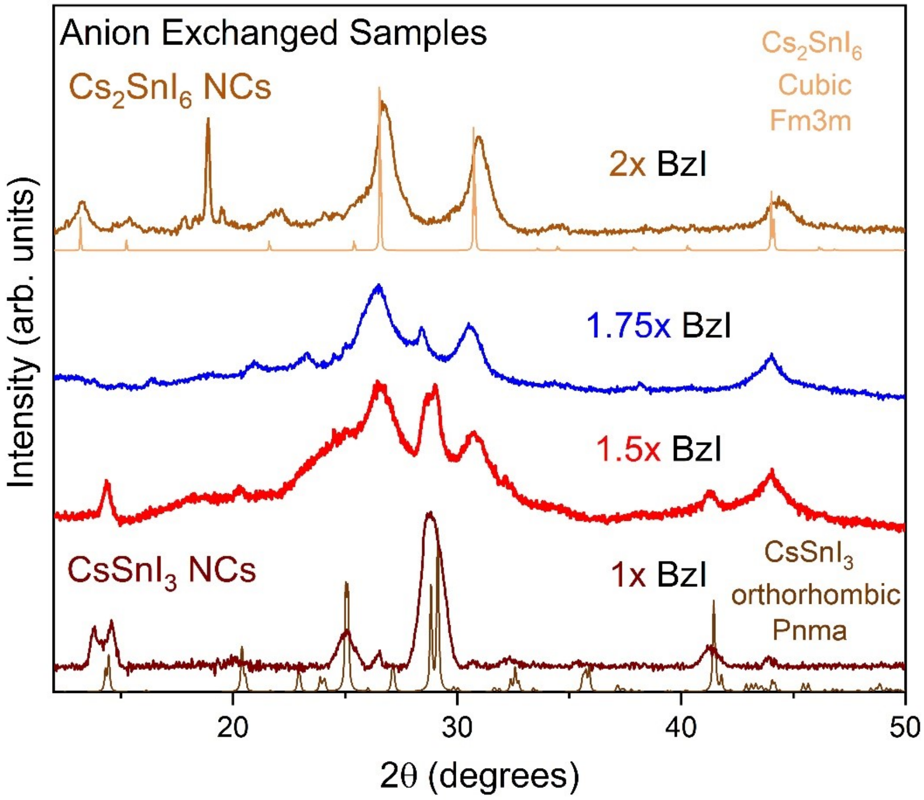

Figure S12. Evolution of powder X-ray diffraction pattern with increasing amount of BzI added for anion exchange. The XRD bulk references are plotted from $CsSnI_3$ (orthorhombic, Pnma)[5] and Cs_2SnI_6 (cubic, Fm3m)[4]

References:

1. M. Morana, J. Wiktor, M. Coduri, R. Chiara, C. Giacobbe, E. L. Bright, F. Ambrosio, F. De Angelis and L. Malavasi, *J. Phys. Chem. Lett.*, 2023, **14**, 2178-2186.
2. D. H. Fabini, G. Laurita, J. S. Bechtel, C. C. Stoumpos, H. A. Evans, A. G. Kontos, Y. S. Raptis, P. Falaras, A. Van der Ven, M. G. Kanatzidis and R. Seshadri, *J. Am. Chem. Soc.*, 2016, **138**, 11820-11832.
3. R. W. G. Wyckoff, *Crystal Structures - Volume 1*, Interscience Publishers New York, New York, 2nd ed edn., 1963.
4. C. C. Stoumpos, C. D. Malliakas and M. G. Kanatzidis, *Inorg. Chem.*, 2013, **52**, 9019-9038.
5. I. Chung, J.-H. Song, J. Im, J. Androulakis, C. D. Malliakas, H. Li, A. J. Freeman, J. T. Kenney and M. G. Kanatzidis, *J. Am. Chem. Soc.*, 2012, **134**, 8579-8587.
